# Bioavailability of Lumefantrine Is Significantly Enhanced with a Novel Formulation Approach, an Outcome from a Randomized, Open-Label Pharmacokinetic Study in Healthy Volunteers

**DOI:** 10.1128/AAC.00868-17

**Published:** 2017-08-24

**Authors:** Jay Prakash Jain, F. Joel Leong, Lan Chen, Sampath Kalluri, Vishal Koradia, Daniel S. Stein, Marie-Christine Wolf, Gangadhar Sunkara, Jagannath Kota

**Affiliations:** a Novartis Healthcare Pvt. Ltd., Hyderabad, India; b Novartis Institute for Tropical Diseases, Singapore; c Novartis Institutes for BioMedical Research, Shanghai, People's Republic of China; d Novartis Pharma, East Hanover, New Jersey, USA; e Novartis Pharma AG, Basel, Switzerland

**Keywords:** malaria, bioavailability, lumefantrine, antimalarial agents, solid dispersion

## Abstract

The artemether-lumefantrine combination requires food intake for the optimal absorption of lumefantrine. In an attempt to enhance the bioavailability of lumefantrine, new solid dispersion formulations (SDF) were developed, and the pharmacokinetics of two SDF variants were assessed in a randomized, open-label, sequential two-part study in healthy volunteers. In part 1, the relative bioavailability of the two SDF variants was compared with that of the conventional formulation after administration of a single dose of 480 mg under fasted conditions in three parallel cohorts. In part 2, the pharmacokinetics of lumefantrine from both SDF variants were evaluated after a single dose of 480 mg under fed conditions and a single dose of 960 mg under fasted conditions. The bioavailability of lumefantrine from SDF variant 1 and variant 2 increased up to ∼48-fold and ∼24-fold, respectively, relative to that of the conventional formulation. Both variants demonstrated a positive food effect and a less than proportional increase in exposure between the 480-mg and 960-mg doses. Most adverse events (AEs) were mild to moderate in severity and not suspected to be related to the study drug. All five drug-related AEs occurred in subjects taking SDF variant 2. No clinically significant treatment-emergent changes in vital signs, electrocardiograms, or laboratory blood assessments were noted. The solid dispersion formulation enhances the lumefantrine bioavailability to a significant extent, and SDF variant 1 is superior to SDF variant 2.

## INTRODUCTION

Malaria continues to have a significant health impact, causing an estimated 438,000 deaths among 214 million cases of malaria worldwide in 2015, and most of these were in sub-Saharan Africa in children less than 5 years of age (1). One of the first-line treatments for falciparum malaria is the combination of lumefantrine and artemether (1). Originally registered in China in 1992, it was subsequently developed by Ciba-Geigy (which became Novartis in 1996) as a fixed-dose combination and registered by Novartis worldwide as artemether-lumefantrine (Coartem or Riamet). Artemether-lumefantrine is now available in over 60 countries and has been used by over 750 million patients (2).

Artemether-lumefantrine is a highly effective and well-tolerated antimalarial with cure rates of >95%, even in areas where the malaria parasites are resistant to multiple other antimalarial drugs (3, 4). Dosing is weight based, and the standard dose is composed of 80 mg artemether and 480 mg lumefantrine and is given twice daily for 3 days. As stated in the prescribing information, artemether-lumefantrine must be administered with high-fat food, since food is known to increase the bioavailability (BA) of lumefantrine by up to 16-fold (5, 6) and that of artemether by up to 3-fold. The administration of artemether-lumefantrine with food is also important to achieve sufficient exposure to lumefantrine up to day 7, which is required for a high cure rate (7, 8). In patients with acute malaria illness and in countries where malaria is endemic, nonadherence to this treatment requirement can lead to treatment failure (9). The overall cure rate for patients receiving artemether-lumefantrine is high, but there is significant variability in the levels of exposure because of differences in food intake, especially in ill patients. A formulation that improves overall exposure with less dependence on food intake could decrease this variability.

Considering the scope for enhancing the oral bioavailability of lumefantrine on the basis of a strong positive food effect, it was postulated that a formulation intervention might achieve higher bioavailability. Hence, two new solid dispersion formulation (SDF) variants of lumefantrine were developed on the basis of preliminary data from studies with dogs under fasted conditions, in which an ∼4-fold increase in BA relative to that of the conventional tablet was observed (Novartis, data on file). The SDF formulations were evaluated for their potential to enhance the BA of lumefantrine under fasting and fed conditions. In addition, the proportionality of a higher dose under fasting conditions was also evaluated.

## RESULTS

### Subject demographics.

A total of 49 male subjects (age range, 18 to 44 years) were randomized into part 1 of the study, and of these, 16 subjects continued into part 2 of the study. The mean body weight for all subjects in each treatment was between 65.7 and 77.4 kg (range, 55.7 to 107.3), and the mean body mass index (BMI) was 21.8 to 23.9 kg/m^2^ (range, 18.2 to 29.8 kg/m^2^). Most of the subjects were Caucasian (51.6%), followed by Asian (37.5%).

### Safety and tolerability.

There were no deaths or serious adverse events (SAEs) reported during this study. In part 1, more adverse events (AEs) were reported with the SDF variants (43.8% each, 7/16 subjects) than with the conventional tablets (18.8%, 3/16 subjects). These included isolated events (postural dizziness, nasal congestion/hypersensitivity, headache, iridocyclitis, and catheter site pain) and respiratory tract infection (the most common AE overall). When upper respiratory tract infections were excluded from the count in part 1, slightly more AEs were associated with SDF variant 2 than with SDF variant 1 (37.5% and 25.0%, respectively). There was no temporal relationship of the respiratory tract infections either with the dose or with its occurrence in the various individuals.

There were 4 AEs in cohort 1 (conventional tablet) affecting three subjects (18.8%): dyspepsia (6.3%, 1/16), gastroenteritis (6.3%), toothache (6.3%), and upper respiratory tract infection (6.3%). Cohort 2 (which received 480 mg of SDF variant 1 under fasting conditions) had 9 AEs affecting 7 subjects (43.8%), with upper respiratory tract infection (25.0%, 4/16) having the highest incidence. Cohort 3 (which received 480 mg of SDF variant 2 under fasting conditions) had 8 AEs affecting 7 subjects (43.8%), with upper respiratory tract infection having the highest incidence (12.5%, 2/16).

Of the 32 AEs reported after dosing, 5 were considered drug related by the investigators. All occurred in subjects taking SDF variant 2, and each occurred in a different subject. There were three in cohort 3, consisting of left anterior uveitis/iridocyclitis (grade 2), nasal congestion (grade 2), postural lightheadedness (grade 1); one in cohort 6 (which received 480 mg of SDF variant 2 under fed conditions), consisting of headache (grade 1); and one in cohort 7 (which received 960 mg of SDF variant 2 under fasting conditions), consisting of fatigue (grade 1).

Two drug-related AEs in cohort 3 (which received 480 mg of SDF variant 2) should be noted, as the two subjects in whom these AEs occurred did not participate in part 2. Only one subject was classified to have discontinued the study, as he left the study on day 48. He was a 22-year-old Caucasian male with no relevant medical history who presented with a 24-h history of left eye itching, eye redness, and photophobia on day 10 and was initially treated with 0.5% chloramphenicol eyedrops for conjunctivitis, a diagnosis which was revised to iridocyclitis/anterior uveitis on day 14 and managed with prednisolone acetate ophthalmic suspension eyedrops and homatropine eyedrops, with full resolution by day 39.

The other subject in whom drug-related AEs occurred was a 21-year-old Caucasian male with a history of hay fever allergy, managed with over-the-counter antihistamines. He did not have any prior exposure to lumefantrine or any other antimalarials or any history of use of any medications or herbal therapies in the days leading up to dosing, and he had no past serious reaction to bee stings. He was dosed with SDF variant 2 on day 1 and experienced symptoms of nasal congestion, facial flushing, dry throat, and a “sensation of wheezing” ∼15 min after dosing. On examination, he was alert and oriented with no stridor and normal vital signs and oxygen saturation. His chest was clear with no evidence of bronchospasm. As a precaution, his symptoms were managed with intravenous hydrocortisone (100 mg) and oral loratadine (10 mg), and full recovery occurred ∼5.5 h after onset. He completed part 1 and by definition did not discontinue, though he was ineligible to participate in part 2 due to his AE. As a precautionary measure, dose scheduling between subjects in part 2 was staggered, with the intent that a study hold would be activated if a similar event occurred again.

In part 2, there were four subjects in each cohort, with two to three subjects in each cohort experiencing an AE. Each AE affected one subject, except for upper respiratory tract infection, which occurred in two subjects. When AEs in the cohorts receiving an SDF in both part 1 and part 2 were pooled, upper respiratory tract infection was unchanged as the most common AE, and there was no temporal relationship to dosing.

Most of the AEs reported were mild to moderate in severity (grade 1 or 2), and most were not suspected to be related to the study drug. No clinically significant treatment-emergent changes in vital signs, electrocardiograms (ECG), or laboratory blood assessments were noted.

### Assessment of PKs.

**FIG 1:**
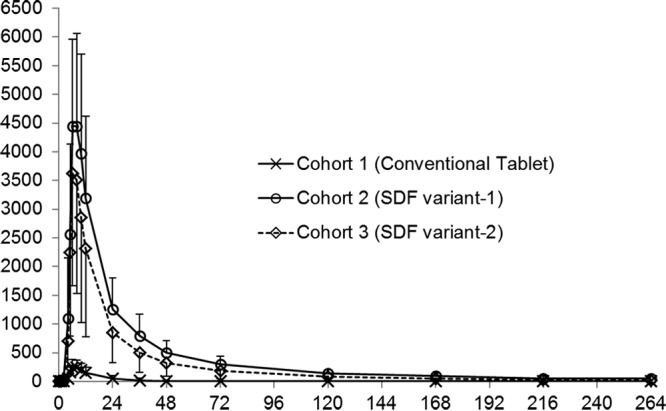
Mean plasma concentration-time profiles of lumefantrine following oral administration of a single 480-mg dose as a conventional tablet, SDF variant 1, and SDF variant 2 under fasting conditions. Cohort 1 received 480 mg of conventional tablets while fasting, cohort 2 received 480 mg of SDF variant 1 capsules while fasting, and cohort 3 received 480 mg of SDF variant 2 capsules while fasting. Error bars indicate ±1 SD. Data on the *y* axis are lumefantrine concentrations (in nanograms per milliliter), and data on the *x* axis are times (in hours).

The concentration-time profiles of lumefantrine following administration of a single dose of 480 mg under fasting conditions as a conventional tablet, SDF variant 1, and SDF variant 2 are presented in Fig. 1. A corresponding summary of the pharmacokinetic (PK) parameters is presented in Table 1. Irrespective of the formulation, lumefantrine was absorbed with a median time to the maximum concentration in plasma (*T*_max_) of 6 h with some initial lag time. On the basis of the measurements obtained up to 11 days postdosing, the terminal elimination half-life (*t*_1/2_) with the SDF formulation was ∼3 to 5 days.

**TABLE 1:** Plasma PK parameters for lumefantrine conventional tablet, SDF variant 1, and SDF variant 2 following oral administration of a single 480-mg dose in the fasted or fed state or a 960-mg dose in the fasted state

PK parameter	Value(s) for cohort:						
	1 (480 mg of conventional tablets, fasting; *n* = 16)	2 (480 mg of SDF variant 1 capsules, fasting; *n* = 16)	3 (480 mg of SDF variant 2 capsules, fasting; *n* = 16)	4 (480 mg of SDF variant 1 capsules, fed; *n* = 4)	5 (960 mg of SDF variant 1 capsules, fasting; *n* = 4)	6 (480 mg of SDF variant 2 capsules, fed; *n* = 4)	7 (960 mg of SDF variant 2 capsules, fasting; *n* = 4)
*C*_max_ (ng/ml)							
Mean ± 1 SD	260 ± 143	4,790 ± 1,680	3,780 ± 2,130	29,700 ± 10,900	8,410 ± 3,970	19,800 ± 346	7,280 ± 2,180
% CV^*a*^	55	35	56.5	36.8	47.2	1.7	30.0
No. of subjects	16	16	16	3	4	3	3
AUC_last_ (μg · h/ml)							
Mean ± 1 SD	3.08 ± 2.66	112 ± 48.9	70.7 ± 50.6	568 ± 183	187 ± 117	477 ± 76.2	121 ± 77.6
% CV	86.2	43.8	71.6	32.3	62.4	16	64.2
No. of subjects	16	13	15	4	3	3	4
AUC_inf_ (μg · h/ml)							
Mean ± 1 SD	7.15 ± 2.27	117 ± 48.6	82.2 ± 50.8	599 ± 206	219 ± 113	496 ± 66.2	129 ± 83.5
% CV	31.7	41.6	61.8	34.3	51.8	13.3	64.8
No. of subjects	6	15	15	4	4	4	4
AUC_0–24_ (μg · h/ml)							
Mean ± 1 SD	2.80 ± 1.89	57.6 ± 22.9	42.8 ± 24.9	290 ± 96.7	107 ± 52.0	261 ± 21.3	71.9 ± 38.9
% CV	67.3	39.7	58.0	33.4	48.8	8.2	54.1
No. of subjects	16	16	16	4	4	4	4
AUC_0–72_ (μg · h/ml)							
Mean ± 1 SD	3.51 ± 2.74	87.1 ± 35.6	61.8 ± 37.3	435 ± 142	162 ± 81.0	388 ± 39.7	104 ± 60.0
% CV	77.9	40.8	60.3	32.7	50.1	10.2	57.7
No. of subjects	16	16	16	4	4	4	4
*T*_max_ (h)							
Median	6	6	6	6	8	8	6
Range	5.00–10.0	6.00–10.0	6.00–10.0	6.00–8.00	6.00–10.0	5.00–8.03	6.00–10.0
No. of subjects	16	16	16	3	4	3	3
*t*_1/2_ (h)							
Mean ± 1 SD	14.2 ± 5.17	76.5 ± 27.3	53.0 ± 38.3	73.7 ± 15.5	115 ± 46.7	94.2 ± 30.2	58.1 ± 44.8
% CV	36.5	35.6	72.2	21.0	40.6	32.0	77.2
No. of subjects	6	15	15	4	4	4	4
CL/*F* (liters/h)							
Mean ± 1 SD	71.9 ± 18.4	5.53 ± 4.72	7.95 ± 4.52	0.869 ± 0.266	5.37 ± 2.60	0.980 ± 0.121	10.9 ± 7.66
% CV	25.5	85.3	56.8	30.6	48.5	12.4	70.3
No. of subjects	6	15	15	4	4	4	4
*V*/*F* (liters)							
Mean ± 1 SD	1,360 ± 259	500 ± 19)	444 ± 120	88.2 ± 12.2	824 ± 366	135 ± 54.3	625 ± 133
% CV	19.0	38.1	27.0	13.8	44.4	40.4	21.3
No. of subjects	6	15	15	4	4	4	4

a CV, coefficient of variation.

The geometric mean ratios and 90% confidence intervals (CIs) for the maximum concentration in plasma (*C*_max_), the area under the concentration-time curve (AUC) from time zero to the time of the last concentration measurement (AUC_last_), and the AUC from time zero to infinity (AUC_inf_) for both SDF variant 1 and SDF variant 2 relative to the conventional tablet (the reference treatment) are presented in Table 2. The rate and extent of absorption of lumefantrine from both of the SDF formulations relative to those of lumefantrine from the conventional formulation were enhanced significantly. The geometric mean *C*_max_ for SDF variant 1 was 4.38 μg/ml. The *C*_max_s for SDF variant 1 and SDF variant 2 were ∼19-fold and ∼13-fold higher, respectively, than the *C*_max_ of the conventional formulation under fasting conditions. The relative bioavailabilities of SDF variant 1 and SDF variant 2 were ∼15- and ∼10-fold higher, respectively, than the relative bioavailability of the conventional formulation under fasting conditions on the basis of the AUC_inf_. Comparison of the level of exposure to the SDF variants with that to the conventional formulation using AUC_inf_ has limited relevance due to the low level of absorption of lumefantrine from the conventional formulation (under fasting conditions), with data from only 6 out of 16 subjects being evaluable. Hence, the comparison was also made using the AUC_last_ values for at least 12 evaluable subjects for each treatment, and the bioavailabilities of SDF variant 1 and SDF variant 2 were ∼48- and ∼24-fold higher, respectively, than the bioavailability of the conventional formulation under fasting conditions.

**TABLE 2:** Geometric mean ratio and 90% CIs for PK parameters^*a*^

PK parameter	Cohort	*n*^*b*^	Adjusted geometric mean value	Cohorts compared	Treatment comparison	
					Geometric mean ratio^*c*^	90% CI
AUC_inf_ (μg · h/ml)	1	6	6.90			
	2	15	104.46	2 vs 1	15.15	9.79, 23.44
	3	15	70.12	3 vs 1	10.17	6.57, 15.73
AUC_last_ (μg · h/ml)	1	16	2.02			
	2	13	97.47	2 vs 1	48.19	26.02, 89.23
	3	15	49.07	3 vs 1	24.26	13.41, 43.90
*C*_max_ (ng/ml)	1	16	224.50			
	2	16	4,376.88	2 vs 1	19.50	13.12, 28.97
	3	16	3,014.73	3 vs 1	13.43	9.04, 19.96

a The log-transformed primary pharmacokinetic parameters *C*_max_, AUC_last_, and AUC_inf_ were analyzed separately by using a linear mixed effects model with treatment (cohort) as the fixed effect. The reference group was cohort 1, which received 480 mg of conventional tablets in the fasting state; cohort 2 received 480 mg of SDF variant 1 capsules in the fasting state; and cohort 3 received 480 mg of SDF variant 2 capsules in the fasting state.

b *n*, number of subjects with nonmissing values.

c The geometric mean ratio was calculated as the test value/reference value.

Under fasting conditions, the *C*_max_ and AUC_inf_ of lumefantrine in the current study were 260 ± 143 ng/ml and 7.15 ± 2.27 μg · h/ml, respectively, which are comparable to historical data (Novartis, data on file), where the reported *C*_max_ and the AUC from time zero to 168 h (AUC_0–168_) (for lumefantrine in combination with artemether) were 383 ± 158 ng/ml and 6.81 ± 3.30 μg · h/ml, respectively. In the same historical study, under fed conditions, *C*_max_ and AUC were 5.1 μg/ml and 107.9 μg · h/ml, respectively, whereas they were 29.7 μg/ml and 568 μg · h/ml, respectively, for SDF variant 1 in this study.

A comparison of the values of the pharmacokinetic parameters was made for the subjects who received the same SDF variant in both part 1 and part 2 of the study. Administration with a high-fat meal resulted in an approximately 8-fold increase in AUC_last_ for both variants, while the increase in *C*_max_ was approximately 12-fold and 6-fold for variant 1 and variant 2, respectively (Table 3).

**TABLE 3:** Geometric mean ratio and 90% CIs for PK parameters to assess food effect

PK parameter	Cohort^*a*^	*n*^*b*^	Adjusted geometric mean value	Cohorts compared	Estimate	
					Geometric mean ratio^*c*^	90% CI
AUC_inf_ (μg · h/ml)	2	4	75.79			
	4	4	574.35	4 vs 2	7.58	3.38, 16.97
	3	4	64.03			
	6	4	492.72	6 vs 3	7.69	6.95, 8.51
AUC_last_ (μg · h/ml)	2	4	68.91			
	4	4	547.28	4 vs 2	7.94	3.21, 19.63
	3	3	62.58			
	6	3	472.78	6 vs 3	7.56	6.49, 8.79
*C*_max_ (ng/ml)	2	3	2,366.87			
	4	3	28,072.73	4 vs 2	11.86	5.26, 26.74
	3	3	3,412.13			
	6	3	19,797.99	6 vs 3	5.80	4.54, 7.42

a Cohort 2 received 480 mg of SDF variant 1 capsules in the fasting state, cohort 3 received 480 mg of SDF variant 2 capsules in the fasting state, cohort 4 received 480 mg SDF of variant 1 capsules in the fed state, and cohort 6 received 480 mg of SDF variant 2 capsules in the fed state.

b *n*, number of subjects with nonmissing values.

c The geometric mean ratio was calculated as the test value/reference value.

Formal proportionality analysis between the 480-mg and 960-mg doses could not be done due to limited data. Nevertheless, the level of exposure for both variants did not increase proportionally with the dose (see Table S1 in the supplemental material). For a 2-fold increase in dose, there were ∼1.5- and ∼1.1-fold increases in the levels of exposure (AUC_inf_) for SDF variant 1 and SDF variant 2, respectively. This should be interpreted with caution, as the data are limited and the 90% confidence interval is wide, especially for SDF variant 2.

## DISCUSSION

Lumefantrine is a highly lipophilic molecule with very limited absorption and bioavailability when administered orally under fasting conditions. It has been classified as a biopharmaceutic (BCS) class II (10) or class IV (11) compound. The very low aqueous solubility of lumefantrine (2.6 ng/ml in fasted state-simulated intestinal fluid [FaSSIF]; Novartis, data on file) could be one of the major reasons for its low bioavailability. This is in agreement with the approximately 16-fold increase in bioavailability when it is administered with high-fat food, potentially due to enhanced *in vivo* solubility (12, 13). Hence, administration with food is essential to achieve the desired exposure (8, 14). The label recommendation (artemether-lumefantrine [Coartem prescribing information]) for administration of artemether-lumefantrine with food or drink rich in fat is principally based on a positive food effect.

Various formulation approaches have been attempted to enhance the bioavailability of lumefantrine (15–22); however, none of these reports presented clinical data. Hence, the goal of this work was to evaluate the SDF variants for their potential enhancement of the bioavailability of lumefantrine in humans.

The outcome of the clinical study demonstrated significantly increased lumefantrine bioavailability with both variants. While the increase in bioavailability was considerably higher (48- and 24-fold, respectively, for SDF variants 1 and 2) than that reported earlier (12, 13) with a high-fat meal (approximately 16-fold), it is important to note that the past study (study COA566A020) did not use the currently defined calorie/fat content for a high-fat meal. In the previous Novartis food effect study (study COA566A020; Novartis, data on file), the lumefantrine conventional formulation was administered with high-calorie food (1,071.1 kcal) with a fat content of only 365.8 kcal, while this study followed current health authority guidance, in which fat consisted of 497 kcal of the 916-kcal meal, which is both an absolute increase and a proportional increase. Hence, it can be assumed that the difference in the fold increase in exposure from the previous study could be due to the lower fat content and the high pharmacokinetic variability of lumefantrine.

The SDF formulations significantly increased the bioavailability of lumefantrine, but there was a further increase with the consumption of food rich in fat, indicating that further increases in bioavailability from formulation enhancements could still be possible. While the food effect arm in the current study claimed a further 8-fold increase in bioavailability with both SDF variants when they were administered with a high-fat meal, the highest concentrations (37.5 μg/ml) achieved in the current study were below the maximum concentrations (60 to 70 μg/ml) achieved with lumefantrine in historical studies with established safety and tolerability (23, 24). Given the additional approximately 8-fold increase in bioavailability of the lumefantrine SDF under fed conditions, it could be postulated that the further increase is due to the enhanced solubility of lumefantrine in the presence of fat, indirectly suggesting that solubility probably plays a critical role in the absorption of lumefantrine after oral administration.

The time to achieve the maximum concentration in plasma (median *T*_max_, 6 h) under both fasting and fed conditions suggests that the absorption site and the absorption mechanism are similar under both conditions. While the mechanism of absorption of lumefantrine has not been systematically evaluated, halofantrine is a molecule similar to lumefantrine with a *T*_max_ of about 6 h (25) and is known to be absorbed via the lymphatic route (26, 27).

Due to data limitations, the proportionality in exposure to 480-mg and 960-mg doses could not be systematically evaluated; however, the observed underproportional increase in the AUC with dose could be empirically considered. For SDF variant 1, the AUC increased by 1.54-fold with a 2-fold increase in dose, and this nonproportional increase in exposure could be attributed to insufficient *in vivo* solubility at the higher doses.

Both SDF variants were generally well tolerated. Overall, there was a higher incidence of AEs in subjects treated with SDF variants (43.8%) than in those treated with the conventional tablets (18.8%), though the majority of AEs were mild and not considered drug related. There was no clear difference in the incidence of adverse events between the two SDF variants. When upper respiratory tract infections were excluded from the count of AEs, there were slightly more AEs associated with variant 2 (37.5% versus 25.0% with variant 1). Additionally, all the drug-related AEs, including the nasal congestion managed as a hypersensitivity reaction, were observed only with SDF variant 2.

The artemether-lumefantrine label mentions hypersensitivity and allergic skin reactions as rare adverse drug reactions (28); however, an allergic reaction solely attributable to lumefantrine has not been reported, probably because lumefantrine is not available as a single agent. Overall, due to the lower incidence of drug-related AEs and its higher bioavailability, SDF variant 1 appears to be the superior formulation for further clinical exploration. Clinical trials to further evaluate the utility of the preferred SDF formulation of lumefantrine in subjects with malaria are planned. The exposures found when the SDF formulation is given both with and without food will be explored further.

## MATERIALS AND METHODS

### Study design.

The study was conducted according to the ethical principles of the Declaration of Helsinki. Informed consent was obtained before randomization from each subject participating in the trial. This study protocol was reviewed and approved by The Alfred Health Ethics Committee (Melbourne, Australia).

This was a randomized, open-label, parallel-group, two-part study conducted in healthy volunteers. Lumefantrine was used as the investigational drug in this study. Lumefantrine SDF variant 1 and SDF variant 2 (capsules of 80-mg strength) were manufactured by Novartis and supplied to the investigator site as an open-label bulk supply.

Due to the long half-life (2 to 6 days) of lumefantrine and the multiple treatments to be assessed, a crossover design was not feasible; therefore, a parallel design was used. An open-label design was justified because the primary endpoint was to assess the relative bioavailability of the same compound. It was thus not confounded by the subjects' or investigators' knowledge of the treatment allocation. The study included a screening period of up to 28 days, two baseline periods (one before each part), and a washout period of a minimum of 5 weeks for subjects who continued to the second part of the study. Subject eligibility evaluation (inclusion/exclusion assessment) was performed during the baseline visit for each study part. Subjects were admitted to the study unit at least 1 day prior to dosing in each period for baseline evaluations.

Forty-eight subjects in part 1 were randomized (1:1:1) to one of the three cohorts (16 subjects in each cohort) receiving a conventional 120-mg lumefantrine tablet (cohort 1), SDF variant 1 consisting of 80-mg capsules (cohort 2), or SDF variant 2 consisting of 80-mg capsules (cohort 3). All subjects were administered a single 480-mg dose under fasting conditions and were followed over 12 days. In part 2, which followed a washout period of 5 weeks, subjects from the SDF variant arms were reallocated to a 480-mg food effect arm or a fasting effect higher-dose arm (960-mg dose) in a 1:1 ratio. After dosing, the subjects were followed for 12 days (days 53 to 64) before a final end-of-study (EOS) visit by day 71 (approximately).

An interim internal review was conducted after approximately 12 subjects from each cohort had completed study visit 108 (day 12) to determine if the lumefantrine SDF variants met the protocol-specified criterion (a >4-fold enhanced lumefantrine bioavailability) to continue into part 2.

For the treatments under fasting conditions, subjects had no food or liquid (except water) for at least 10 h prior to administration of study drug and continued to fast for at least 4 h postdosing. For the treatments under fed conditions, subjects were provided a high-fat breakfast (a total of 916 cal with 178, 241, and 497 cal from protein, carbohydrate, and fat, respectively). The meal was served and consumed within 30 min, and the study drug was administered within 5 min after completion of the meal. All doses were administered with 180 to 240 ml water.

### Subjects.

The eligible study population comprised healthy males or females of nonchildbearing potential in the age range of 18 to 55 years and of at least 50 kg in weight (BMI, within 18.0 to 30.0 kg/m^2^).

The main exclusion criteria were pregnancy or nursing for women, smoking, a medical history of clinically significant cardiac or ECG abnormalities (including arrhythmias), a family history of a prolonged QT interval syndrome, or the receipt of drugs that are able to alter the activity of the CYP3A4 enzyme or that are CYP2D6 substrates within 4 weeks or 5 times the terminal half-life of each drug prior to dosing.

Subjects could voluntarily withdraw from the study for any reason at any time or be withdrawn for safety reasons. They were considered withdrawn if they stated an intention to withdraw, failed to return for visits, or became lost to follow-up for any other reason. Once the minimum number of subjects (12 per cohort) was achieved, there were no replacements.

### Treatments.

Three treatments were used in this study: a conventional tablet with lumefantrine at 120 mg (the reference formulation), 80-mg capsules of lumefantrine SDF variant 1, and 80-mg capsules of lumefantrine SDF variant 2.

The solid dispersion formulations contained lumefantrine in an amorphous form for potential enhancement of solubility and oral bioavailability, whereas lumefantrine in the conventional tablet formulation is in a stable crystalline form. Various approaches to the preparation of a solid dispersion of lumefantrine have been reported (29–31), but they have not been tested in human studies to evaluate if they improve bioavailability. The solid dispersion variants included excipients to stabilize the amorphous form, and SDF variant 2 contains some additional surfactant. The solid dispersion blend produced by hot melt extrusion was further mixed with fillers, disintegrants, and lubricants so that it could be formulated into a capsule dosage form. Each capsule contained 80 mg of lumefantrine along with other excipients. In comparison, the standard tablet formulation was manufactured by a standard process of mixing, wet granulation (standard technology), milling, blending, and compression.

### Safety assessment.

Safety assessments included evaluations of vital signs, ECGs, and clinical laboratory assessment values. All data were listed by treatment group, subject, and visit/time, and abnormalities were reviewed. The Common Terminology Criteria for Adverse Events (CTCAE; v4.03) was used to grade the adverse events and abnormalities. All information on adverse events obtained was displayed by treatment group (and subject). The number and percentage of subjects with adverse events were tabulated by preferred term or by body system with a breakdown by treatment (cohort). Multiple adverse events within a body system of a subject were counted only once toward the total for that body system.

### Assessment of PKs.

All blood samples were taken either by direct venipuncture or via an indwelling cannula inserted in a forearm vein. Samples were obtained for pharmacokinetic (PK) analysis, and PKs were evaluated in all subjects at all dose levels. Blood samples for PK analysis of lumefantrine were collected predosing and at 1, 2, 4, 5, 6, 8, 10, 12, 24, 36, 48, 72, 120, 168, 216, and 264 h postdosing.

Concentrations below the lower limit of quantitation were considered zero for the pharmacokinetic analysis. The following pharmacokinetic parameters were determined from the plasma concentration-time data by noncompartmental analysis in Phoenix WinNonlin software (v6.4): *C*_max_, *T*_max_, AUC from time zero to 72 h (AUC_0–72_), AUC_last_, AUC_inf_, *t*_1/2_, volume of distribution (*V*/*F*), and clearance (CL/*F*). The linear trapezoidal rule was used for calculation of AUCs.

### Statistical methods.

In part 1, a sample size with 36 subjects (12 per cohort) with complete data from each cohort would allow adequate detection of at least a 1.5-fold change. For the ratios observed from a 1.5-fold to a 5.0-fold change, the predicted 90% confidence intervals for the ratio for the primary pharmacokinetic parameters (AUC_inf_, AUC_last_, and *C*_max_, based on log transformation), determined using the historic data on variability (32), were as follows: 1.08 to 2.09 for a 1.5-fold change, 1.44 to 2.78 for a 2.0-fold change, 2.15 to 4.18 for a 3.0-fold change, 2.87 to 5.57 for a 4.0-fold change, and 3.59 to 6.96 for a 5.0-fold change. In part 2, no formal statistical calculations were considered in calculation of the sample size.

The log-transformed primary pharmacokinetic parameters (*C*_max_, AUC_last_, and AUC_inf_) were analyzed separately by using a linear effects model with treatment as the fixed effect. The estimated mean and 90% confidence intervals of the differences in the treatments were back transformed to obtain the geometric mean ratio and 90% confidence intervals of the geometric mean ratio, and these were reported to represent the relative bioavailability of SDF variant 1 versus that of the conventional tablet and the relative bioavailability of SDF variant 2 versus that of the conventional tablet.

To assess the pharmacokinetics of the higher single dose of 960 mg in comparison to the single dose of 480 mg, log-transformed values of the primary pharmacokinetic parameters (*C*_max_, AUC_last_, and AUC_inf_) were compared using a fixed effects model with treatment and subject as the fixed effects. The estimated mean and 90% confidence intervals of the differences in the treatments were back transformed to obtain the geometric mean ratio and 90% confidence intervals of the geometric mean ratio, and these were reported to represent the exposure of an SDF(s) at the 960-mg single-dose strength, using the results obtained with the 480-mg dose as a reference.

An exploratory assessment of the effect of food between treatments in the fasted and fed states was evaluated for the log-transformed values of the primary pharmacokinetic parameters (*C*_max_, AUC_last_, and AUC_inf_) using a fixed effects model with treatment and subject as the fixed effects. The estimated mean and 90% confidence intervals of the differences in the treatments were back transformed to obtain the geometric mean ratio and 90% confidence intervals of the geometric mean ratio, and these were reported to represent the bioavailability under fed conditions relative to that under fasted conditions.

## Supplementary Material

Supplemental material
