# Supplementary material for: Bioavailability of Lumefantrine Is Significantly Enhanced with a Novel Formulation Approach, an Outcome from a Randomized, Open-Label Pharmacokinetic Study in Healthy Volunteers
Source: Antimicrob Agents Chemother. 2017 Aug 24;61(9):e00868-17. doi: 10.1128/AAC.00868-17 (PMC5571342; doi:10.1128/AAC.00868-17)
Supplement: Supplemental material [file supp_61_9_e00868-17__index.html]

Supplemental material 

# Bioavailability of Lumefantrine Is Significantly Enhanced with a Novel Formulation Approach, an Outcome from a Randomized, Open-Label Pharmacokinetic Study in Healthy Volunteers

## Supplemental material

- Supplemental file 1 -

  Table S1 and Text S1

  PDF, 199K
