# Supplementary material for: Bioavailability of Lumefantrine Is Significantly Enhanced with a Novel Formulation Approach, an Outcome from a Randomized, Open-Label Pharmacokinetic Study in Healthy Volunteers
Source: Antimicrob Agents Chemother. 2017 Aug 24;61(9):e00868-17. doi: 10.1128/AAC.00868-17 (PMC5571342; doi:10.1128/AAC.00868-17)
Supplement: Supplemental material [file AAC.00868-17_zac009176461s1.pdf]

Bioavailability of lumefantrine is significantly enhanced with a novel formulation approach: A randomized, open-label pharmacokinetic study in healthy volunteers

Running title: Novel solid dispersion formulations of lumefantrine

Jay Prakash Jain[1#]*, F. Joel Leong[2#]*, Lan Chen[3], Sampath Kalluri[1], Vishal Koradia[5], Daniel S. Stein[4], Marie-Christine Wolf[5], Gangadhar Sunkara[4], Jagannath Kota[1]

1. Novartis Healthcare Pvt Ltd, Hyderabad, India;
2. Novartis Institute for Tropical Diseases, Singapore;
3. Novartis Institutes for BioMedical Research, Shanghai, PR China;
4. Novartis Pharma, East Hanover NJ, USA;
5. Novartis Pharma AG, Basel, Switzerland;

*These authors contributed equally to this work
#Correspondence to: Joel Leong, joel.leong@novartis.com and Jay Prakash Jain, jay_prakash.jain@novartis.com

Supplementary data

Table S1 Geometric mean ratio (test/reference) and 90 percent confidence intervals for pharmacokinetic parameters assessing a 960 mg versus 480 mg lumefantrine dose

					Estimates	
Parameter	Treatment	N*	Adjusted Geo-mean	Comparison	Geo-mean ratio	(90% CI)
AUCinf (h×µg/mL)	Cohort 2	4	128.05			
	Cohort 5	4	197.28	Cohort 5 vs Cohort 2	1.54	(1.15, 2.06)
	Cohort 3	4	96.25			
	Cohort 7	4	107.18	Cohort 7 vs Cohort 3	1.11	(0.41, 3.02)
AUClast (h×µg/mL)	Cohort 2	3	108.92			
	Cohort 5	3	165.96	Cohort 5 vs Cohort 2	1.52	(0.96, 2.41)
	Cohort 3	3	87.56			
	Cohort 7	3	111.21	Cohort 7 vs Cohort 3	1.27	(0.23, 6.97)
Cmax (ng/mL)	Cohort 2	4	5250.56			
	Cohort 5	4	7619.35	Cohort 5 vs Cohort 2	1.45	(1.17, 1.79)
	Cohort 3	3	5550.34			
	Cohort 7	3	7025.31	Cohort 7 vs Cohort 3	1.27	(0.42, 3.83)

n* = number of subjects with non-missing values

Treatment- Cohort 2: 480 mg SDF variant-1 capsules (Fasting); Cohort 3: 480 mg SDF variant-2 capsules (Fasting); Cohort 5: 960 mg SDF variant-1 capsules (Fasting); Cohort 7: 960 mg SDF variant-2 capsules (Fasting)

Liquid chromatography-Mass spectrometry (LC-MS/MS) method

Lumefantrine concentration was determined by a validated Liquid chromatography-Mass spectrometry (LC-MS/MS) method with a Lower Limit of Quantification (LLOQ) of 50 ng/mL. Briefly, the bioanalytical method consisted of protein precipitation followed by solid phase extraction of human plasma samples and analysis of diluted samples by LC-MS/MS in Multiple Reaction Monitoring (MRM) positive mode using Electrospray Ionization (ESI) as the ionization technique. The lower and upper limits of quantification for linear range were 50.0 ng/mL and 20000 ng/mL respectively using 10 µL of human plasma. Lumefantrine in human plasma is stable for 44 hours at room temperature; 9 months at ≤ -70°C; 3 freeze/thaw cycles at ≤ -70°C; the extract is stable for 100 hours in an autosampler at 8°C. The stability data could cover the period from sampling to analysis of all study samples. For eight points calibration concentration (50, 100, 200, 500, 2000, 5000, 16000 and 20000 ng/mL) bias was within the range of ±15.0% at all

concentrations except for LLOQ (50 ng/mL) for which it was within the range of ±20.0%. For quality control samples (150 ng/mL, 2500 ng/mL and 15000 ng/mL) bias was within the range of ±15.0% for at least 2/3 of the individual values.
